# Distinct neurexin isoforms cooperate to initiate and maintain foraging activity

**DOI:** 10.1038/s41398-023-02668-z

**Published:** 2023-11-30

**Authors:** Brandon L. Bastien, Mara H. Cowen, Michael P. Hart

**Affiliations:** Department of Genetics, University of Pennsylvania, Philadelphia, PA 19104 USA

**Keywords:** Molecular neuroscience, Physiology

## Abstract

Neurexins are synaptic adhesion molecules that play diverse roles in synaptic development, function, maintenance, and plasticity. Neurexin genes have been associated with changes in human behavior, where variants in *NRXN1* are associated with autism, schizophrenia, and Tourette syndrome. While *NRXN1, NRXN2*, and *NRXN3* all encode major α and β isoforms, *NRXN1* uniquely encodes a γ isoform, for which mechanistic roles in behavior have yet to be defined. Here, we show that both α and γ isoforms of neurexin/*nrx-1* are required for the *C. elegans* behavioral response to food deprivation, a sustained period of hyperactivity upon food loss. We find that the γ isoform regulates initiation and the α isoform regulates maintenance of the behavioral response to food deprivation, demonstrating cooperative function of multiple *nrx-1* isoforms in regulating a sustained behavior. The γ isoform alters monoamine signaling via octopamine, relies on specific expression of NRX-1 isoforms throughout the relevant circuit, and is independent of neuroligin/*nlg-1*, the canonical trans-synaptic partner of *nrx-1*. The α isoform regulates the pre-synaptic structure of the octopamine producing RIC neuron and its maintenance role is conditional on neuroligin/*nlg-1*. Collectively, these results demonstrate that neurexin isoforms can have separate behavioral roles and act cooperatively across neuronal circuits to modify behavior, highlighting the need to directly analyze and consider all isoforms when defining the contribution of neurexins to behavior.

## Introduction

**Fig. 1:**
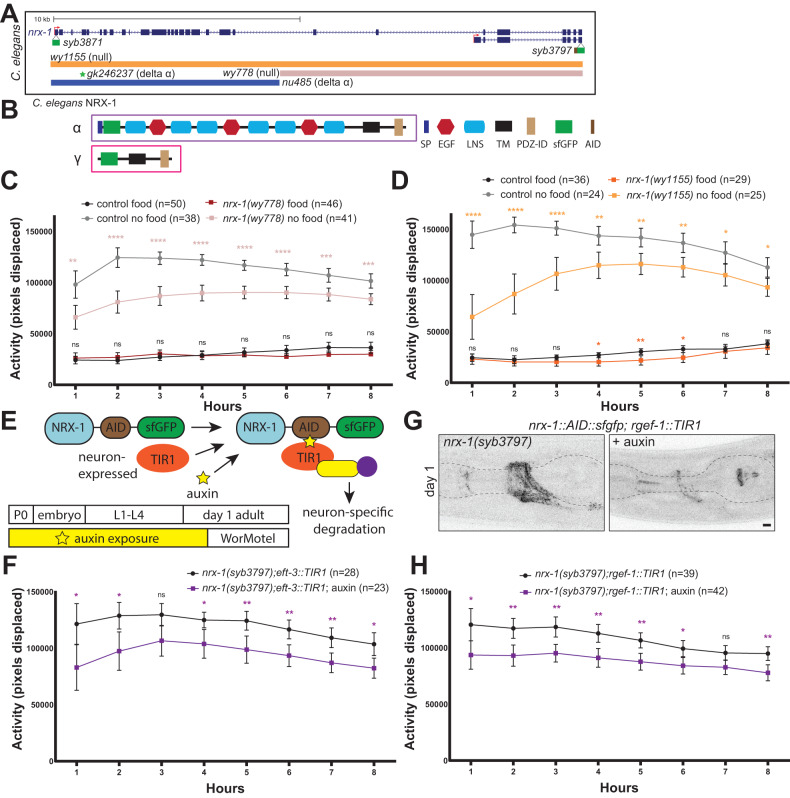
*nrx-1* contributes to the behavioral response to food deprivation in neurons. **A** Schematic of the *nrx-1* gene and alleles used in this study. **B** Cartoon of protein domains for α (purple) and γ (pink) NRX-1 isoforms (SP signal peptide, EGF epidermal growth factor like, LNS laminin /neurexin/sex hormone-binding globulin, TM transmembrane, PDZ-ID PDZ interaction domain, sfGFP superfolder green fluorescent protein, AID auxin inducible degron tag). Average activity of day 1 adult control hermaphrodites in the presence (black) or absence of food (gray) compared with average activity of (**C**) *nrx-1(wy778)* worms (red with food, rose without food), (**D**) *nrx-1(wy1155)* worms (dark orange with food, light orange without food). **E** Cartoon of auxin-inducible degron (AID) and superfolder GFP (sfGFP) tag on all isoforms (*nrx-1(syb3797)*). Combination of this endogenous insertion with auxin and neuron-expressed TIR1 results in NRX-1 degradation in neurons. Timeline of auxin exposure from P0 parent through day 1 adult when behavior was conducted on WorMotel. Average activity of day 1 adult hermaphrodites off food with AID tagged NRX-1 expressing TIR1 in either (**F**) all somatic cells (*eft-2::*TIR1) or (**H**) all neurons (*rgef-1::TIR1)* raised without (black) or with (purple) auxin. **G** Representative confocal micrographs of sfGFP and AID tagged NRX-1 with TIR1 expression in neurons (*rgef-1::TIR1)* raised without (left) and with (right) 4 mM auxin (pseudo-colored black/white). Control worms for each panel represent those run with each allele respectively. Means are plotted with 95% confidence intervals. Results of the Two-way ANOVA with Tukey HSD or a Šidák post hoc test: ns *p* > 0.05, *p < 0.05, **p < 0.01, ***p < 0.001, ****p < 0.0001.

Behavior requires coordination of structural and functional properties of neuronal circuits. Synaptic adhesion molecules (SAMs) facilitate the wiring of neuronal circuits by connecting neurons at synapses and conferring other functional properties by modifying synapse structure and function. Neurexins are a family of SAMs that mediate synapse specification, maturation, maintenance, and plasticity [1]. Humans have three neurexin genes (*NRXN1, NRXN2, and NRXN3*) that each encode major α and β isoforms, while *NRXN1* encodes a recently discovered γ isoform [2–4]. Additionally, multiple alternative splice sites result in the generation of hundreds of isoforms from each gene that are differentially expressed [5–8] (Fig. 1A). Individual isoforms can regulate distinct protein and molecular interactions conferring unique synaptic functionality [2, 3, 9–12]. Mutations and copy number variants in neurexins are associated with characteristic changes in human behavior [13], and variation in the *NRXN1* gene is genetically associated with autism spectrum, Tourette syndrome, schizophrenia, developmental delay, intellectual disability, obsessive compulsive disorder, epilepsy, attention deficit and hyperactivity disorder, and Pitt Hopkins Syndrome 2 [13–16]. These *NRXN1* associations highlight the importance of neurexins in brain function and vulnerability of circuits and behaviors to *NRXN1* perturbation.

Research in animal models has identified molecular functions for neurexins at synapses and in behavior. Loss of the α isoform of *Nrxn1* in mice alters grooming, nest-building, and motor learning behaviors, and possibly anxiety and social behaviors [17, 18]. Transgenic mice expressing mutant *Nrxn1(*β) display modified social and olfactory behaviors [19], while conditional knockout of β neurexins impaired contextual fear memory [9]. Loss of *Nrxn1(*α) in rats impairs reward learning [20], while conditional deletion of *Nrxn1(*α) in excitatory neurons alters value-based operant tasks and reinforcement learning [21]. Flies lacking *dnrx*, which only encodes an α isoform, have altered synapses [22, 23], metabolism [24], and behavior, including sleep, associative learning, and seizures [24–26]. However, the behavioral functions of neurexins lack direct comparison between gene and isoform manipulations and behavioral paradigms, leaving many gaps in our knowledge about isoform-specific roles of neurexin genes in behavior [13, 27–29].

*C. elegans* have a single neurexin gene (*nrx-1*) that encodes α and γ isoforms (Fig. 1A, B), study of which allowed novel discovery of molecular functions including synaptic retrograde signaling [30, 31], specific receptor clustering, synapse and spine formation [31–33], and synaptic partner specificity [33, 34]. *C. elegans* provided the first mechanistic insight into γ neurexin, finding that it promotes synaptic transmission of a cholinergic motor neuron through assembly and recruitment of active zone components and calcium channels, and impacts functional connectivity measured by evoked tail flexion [35]. A pre-synaptic organizational role for the γ isoform was also recently confirmed in mammalian cell culture [2]. Studies of the synaptic functions of *nrx-1* rarely extend to behavior [36, 37], and behavioral studies rarely analyze roles for distinct isoforms [38, 39]. The significant progress in identifying synaptic and circuit roles for neurexin isoforms has left open ties to behavior, especially for the γ isoform [40].

Foraging is a conserved behavior across all animals and *C. elegans* have well-defined behavioral responses to food deprivation. The neuronal circuit involved in the response to food and food deprivation in *C. elegans* includes monoamine signaling from a pair of octopamine-producing interneurons (RIC) [41, 42]. The role of octopamine signaling in activity levels is conserved in other invertebrates, including *Drosophila melanogaster* [43], and is comparable to vertebrate norepinephrine. Here, we use the WorMotel device to monitor individual *C. elegans* to quantify differences in activity in response to food deprivation (Suppl. Fig. 1A). We identify distinct and cooperative roles for the α and γ isoforms of *nrx-1* in the behavioral response to food deprivation. We discover that the γ isoform of *nrx-1* is required for initiation of the response to food deprivation, involving octopamine signaling, while the α isoform of *nrx-1* is required for maintenance of the behavioral response, involving an α isoform-specific role in RIC neuron synaptic structure, and genetic interaction with *nlg-1*.

## Methods

### *C. elegans* strains

*C. elegans* were grown on Nematode Growth Media (NGM) plates seeded with bacteria (*Escherichia coli* OP50) for food and kept at 20–23 °C. For auxin-induced degradation experiments, P0 parents were grown on OP50 seeded NGM plates with 4 mM auxin (Indole-3-acetic acid, ChemProducts), with auxin exposure throughout the development and life. The N2 Bristol strain or OH15098 strains are the control strains for all experiments as noted (OH15098 controls for *him-8(e1489)* and *otIs525* transgene). All strains are listed in Supplemental Table 1. Plasmids and co-injection markers were injected at 20 ng µl^−1^ to generate extrachromosomal arrays. For extrachromosomal transgenes, multiple independent transgenic lines were initially generated and analyzed to confirm initial expression and behavioral phenotypes after which a single line was selected for comprehensive analysis based on consistency of expression levels [44]. *nrx-1*(*syb3797)* and *nrx-1(syb3871*) were generated via CRISPR/cas9 by SunyBiotech and used to make strains MPH10, MPH12, MPH13, MPH32, MPH33.

### Cloning

All plasmids are listed in Supplemental Table 2. To generate pMPH34 (*ric-19::sfGFP::nrx-1(α)*), superfolder GFP cDNA was subcloned into pMPH24 [36] replacing the BirA tag. To generate pMPH35 (*ric-19::sfGFP::nrx-1(*γ*)*), m isoform cDNA of *nrx-1* was subcloned into pMPH34, replacing the full-length α isoform *nrx-1* cDNA. To generate pMPH36, pMPH37, and pMPH38, the *tbh-1* promoter was subcloned into pMPH34 and pMPH35 to replace the *ric-19* promoter using primers indicated in Supplemental Table 2. pMPH38 (*tbh-1p::cla-1::gfp*) was generated by subcloning the *tbh-1* promoter to replace the *lim-6*^*int4*^ promoter in pMPH21 [36].

### WorMotel behavioral assays for food deprivation and octopamine response

WorMotels were fabricated by molding PDMS in a 3D-printed master [41, 45]. WorMotel devices consist of 48 wells, each 3 mm in diameter and depth, and separated by a 3 mm deep moat. Chips were made temporarily hydrophilic via treatment with plasma oxygen with RF settings set to medium (Basic Plasma Cleaner PDC-32g, Harrick Plasma) and each well filled with ~8 µL of melted NGM agar. In conditions with food, or with food and octopamine, 1.5 µL of concentrated OP50 *E. coli* was pipetted onto the well. Exogenous octopamine (Alfa Aesar, J61281) dissolved in concentrated OP50 was applied at 25 mg/mL. WorMotels were dried in a 10 cm (Parter, 1158J69) petri dish with a damp tissue to maintain humidity.

*C. elegans* were collected as late L4s and grown to day 1 young adults (~5 h after collection) on OP50, then washed with M9 solution to remove residual food. WorMotels were loaded by pipetting individual animals in M9 buffer into wells (a) without food, or (b) onto a plate and, once dried, placed manually onto a well with food to avoid food displacement on the WorMotel. The lid to the petri dish was coated with 20% Tween 20 (Fisher BP337-500) to reduce condensation and the petri dish with the WorMotel and damp tissue was placed in a WormWatcher imaging platform (Tau Scientific Instruments, West Berlin, NJ, USA).

WormWatcher imaging platforms were used to capture images every ten seconds for 8 h with a 12-megapixel machine vision camera (Basler Ace U acA4024-8gm) with a 75 mm focal length, f/3.9 high resolution lens (Tamron 23FM75-L). Images for the first 8 h were used for food deprivation experiments and the first 4 h were used for octopamine experiments. Images were processed using a previously reported custom MATLAB code and frame subtraction method [46, 47] that segments individual wells and consecutive images undergo pixel subtraction to calculate the activity by change in pixels. The total pixels displaced over a 1-h period was calculated for each well. Before analysis, the hourly data was checked for inconsistent values (such as large drops in activity from 1 h to the next), prompting manual inspection of images for escaped animals then not included in the analysis. Data from multiple independent replicates were pooled to ensure sufficient power.

### Food finding assay

To test foraging ability, 10 μL of OP50 *Escherichia coli* food was pipetted in the center of a 10 cm petri dish filled with standard NGM agar, allowed to dry, and repeated to create a food target area. 8 well fed day 1 adults were placed equidistant from each other along the rim of the dish ~4.5 cm away from the food and monitored for a duration of 3 h (10800 s). The time of first contact with the food target was recorded for each individual, which were immediately removed and recorded as finishers, while those that did not, were recorded as non-finishers and assigned a time of 10800 s.

### Microscopy

*C. elegans* were anesthetized using 5 µL of 100 mM of sodium azide (NaN_3_) on a pad of 5% agarose on glass slides and covered with a glass coverslip and analyzed using an inverted Leica TCS SP8 laser-scanning microscope operated by LAS X software. Identical laser and photomultiplier tube settings were used for image acquisition across experimental conditions. Confocal micrographs were generated by compressing z-stacks as maximum intensity projections in FIJI.

#### *cla-1::gfp* puncta analysis

Confocal z-stacks were opened, and a threshold filter applied and analyzed using the FIJI 3D objects counter. The number of puncta was verified manually to ensure consistency between the observer and the analysis software. 3D objects counter analysis calculated volume for each puncta, which was summed to compare total volume and divided by number of puncta to compare average puncta volume. Figures were prepared using Adobe Photoshop CS6 and Adobe Illustrator CS6.

### Statistics and reproducibility

Activity values of food deprivation WorMotels were binned into individual hours to measure changes in foraging responses over time and analyzed using a two-way repeated measures ANOVA with a Tukey HSD or a Šidák post-hoc to compare food and no food conditions of all genotypes with each other at each time point. Sphericity was not assumed, so a Greenhouse-Geisser correction was applied. Activity values of the first 4 h of octopamine treatment WorMotels were combined and analyzed using a one-way ANOVA with a Tukey HSD post-hoc to compare all conditions with each other. *cla-1* puncta data was analyzed using a one-way ANOVA with a Tukey HSD post-hoc to compare control and mutant puncta number. Percentage of worms that found the food were compared with a Pairwise Fisher’s Exact tests and the proportion of worms that found the food over time were compared with a pairwise log rank test, both with Bonferroni correction applied. The latency to reach the food in the food finding assay was analyzed using a one-way ANOVA and Tukey HSD post-hoc. Statistical analyses were conducted in Prism and all graphs were generated using Prism. Required sample size was calculated via a power analysis conducted using G*Power [48]. All genotypes had at least one WorMotel experiment where they were placed on a given row to address any well location effect, therefore, animals were not randomized and the experimenter was not blinded. However, the unbiased computer vision analysis determined the activity values for WorMotels. The number of individuals/biological replicates are listed in the figures.

## Results

### *nrx-1* is required in neurons for the *C. elegans* behavioral response to food deprivation

To define the role of *nrx-1* in the behavioral response to food deprivation, we monitored activity of individual *C. elegans* with and without food (Suppl. Fig. 1A) and tested two *nrx-1* deletion alleles that disrupt both α and γ isoforms and are phenotypic nulls (*wy778* and *wy1155*) (Fig. 1A). As previously reported, control animals robustly increase locomotion/activity in wells without food compared to wells with food (Fig. 1) [41]. *nrx-1* null mutants without food had significantly reduced activity compared to controls for all 8 h (Fig. 1C, D, Suppl. Fig. 1B, Suppl. Fig. 2A, B). *nrx-1* is expressed in most or all neurons and other non-neuronal tissues [49]. In order to degrade endogenous NRX-1 with spatial specificity, an auxin inducible degron (AID) and superfolder green fluorescent protein (sfGFP) tag were inserted into the shared C-terminus of *nrx-1* isoforms, just before the PDZ interaction domain (*syb3797*, Fig. 1A). This insertion did not impact the response to food deprivation and does not disrupt NRX-1 function (Suppl. Fig. 1C). We degraded NRX-1 by expressing the TIR1 F-box protein under cell-type specific promoters and exposure to auxin on agarose plates (Fig. 1E). Auxin did not impact the response to food deprivation (Suppl. Fig. 1D). Degradation of endogenous NRX-1 in all somatic cells throughout development (*eft-3p::tir1*) reduced the response to food deprivation compared to controls (Suppl. Fig. 1E, Fig. 1F), similar to *nrx-1* null alleles. Degradation of endogenous NRX-1 specifically in neurons (*rgef-1p::tir1*) similarly reduced activity off food (Fig. 1G, H, Suppl. Fig. 1F), suggesting a primarily neuronal function for *nrx-1* in the behavioral response to food deprivation.

### Both *nrx-1* isoforms contribute to the response to food deprivation

**Fig. 2:**
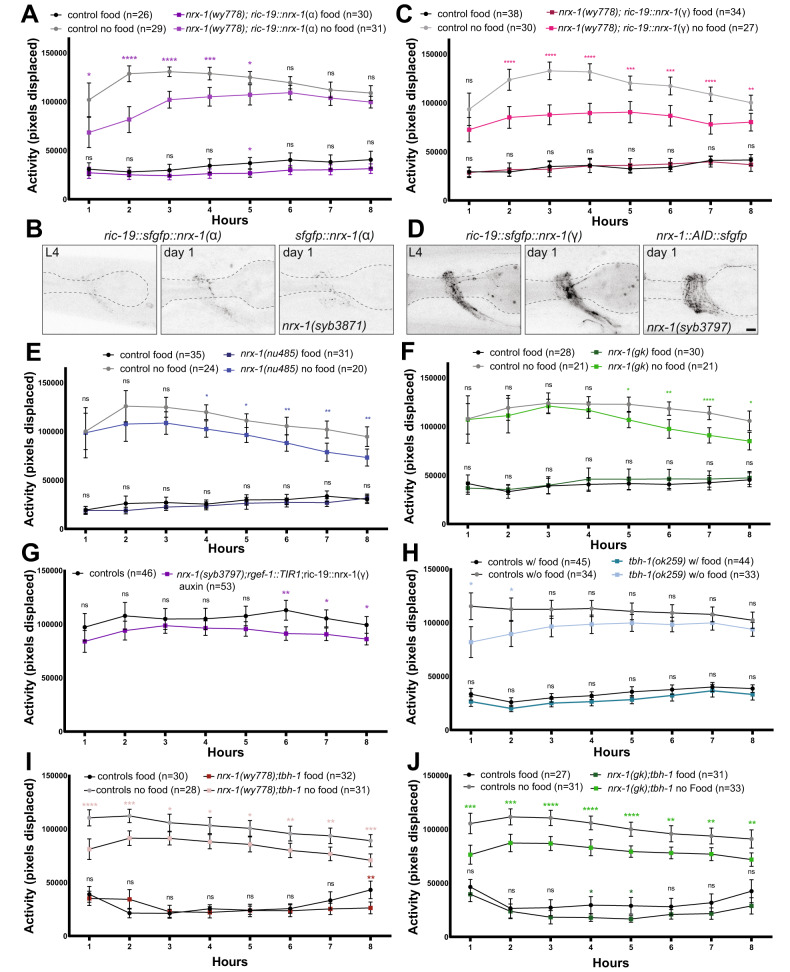
Both *nrx-1* isoforms contribute to the behavioral response to food deprivation. **A** Average activity of control worms in the presence (black) or absence of food (gray) compared to *nrx-1(wy778)* mutant worms expressing α NRX-1 isoform in all neurons (*ric-19* promoter)(dark purple with food, light purple without food). Confocal micrographs of sfGFP tagged NRX-1 (pseudo-colored black/white) in (**B**) L4 or adult *nrx-1(wy778)* mutants expressing α NRX-1 in all neurons compared to endogenous sfGFP tagged α NRX-1 and (**D**) L4 or adult *nrx-1(wy778)* mutants expressing γ NRX-1 in all neurons compared to endogenous sfGFP tagged NRX-1(all isoforms)(scale bar = 10 μm). **C** *nrx-1(wy778)* mutant worms expressing the γ NRX-1 in all neurons (*ric-19* promoter)(dark pink with food, light pink without food). Average activity of control worms in the presence (black) or absence of food (gray) compared to the average activity of (**E**) *nrx-1(nu485)* mutants (dark blue with food, light blue without food), and (**F**) *nrx-1(gk)* mutants (dark green with food, light green without food). **G** Average activity of day 1 adult hermaphrodites with AID and sfGFP tagged NRX-1 and TIR1 in neurons (*rgef-1::TIR1)* also expressing the γ isoform of *nrx-1* in all neurons (*ric-19* promoter) raised without (black) or with (purple) auxin. **H** Average activity of control worms in the presence (black) or absence of food (gray) compared to the average activity of *tbh-1* mutant worms (dark blue with food, light blue without food). **I** Average activity of day 1 adult control hermaphrodites in the presence (black) or absence of food (gray) compared with average activity of *nrx-1(wy778);tbh-1* mutants (red with food, rose without food). **J** Average activity of day 1 adult control hermaphrodites in the presence (black) or absence of food (gray) compared with average activity of *nrx-1(gk);tbh-1* mutants (dark green with food, light green without food). Control worms for each panel represent those run with each mutant allele respectively. Means are plotted with 95% confidence intervals. Results of the Two-way ANOVA with Tukey HSD or a Šidák post hoc test: ns *p* > 0.05, *p < 0.05, **p < 0.01, ***p < 0.001, ****p < 0.0001.

To confirm and further explore the neuronal role of *nrx-1*, we expressed the full-length (α-isoform) cDNA of *nrx-1* in all neurons (*ric-19* promoter) tagged at the N-terminus with sfGFP (Fig. 2A) [50–52]. *nrx-1* mutants expressing NRX-1(α) in all neurons had reduced activity at early time points, but not later time points, compared to controls off food (Fig. 2A, Suppl. Fig. 3A). We observed punctate expression of transgenic NRX-1(α) in the nerve ring and nerve cords at L4 and day 1, likely representing pre-synaptic puncta (Fig. 2B). This expression of NRX-1(α) is well before the behavioral assay, and although it may include ectopic expression of NRX-1, the levels and localization are comparable to endogenous NRX-1(α)(*syb3871)*(Fig. 1A, Fig. 2B). Our results suggest NRX-1(α) functions in neurons to mediate later timepoints of the behavioral response to food deprivation. The lack of rescue at early timepoints may suggest *nrx-1* is required in non-neuronal tissues [30], a specific subset of neurons [34], or in an isoform-specific manner [35, 37]. Expression of the γ isoform of *nrx-1* tagged at the N-terminus with sfGFP in neurons (*ric-19*p) resulted in similar activity off food compared to controls during the first hour of food deprivation, but reduced activity at later timepoints (Fig. 2C, Suppl. Fig. 3B). Transgenic expression of NRX-1(γ) in all neurons appeared punctate in the nerve ring and cords with some neurites showing diffuse localization (Fig. 2D). The expression of NRX-1(γ) was comparable to the levels of endogenous NRX-1 where all isoforms were tagged (*syb3797*), although there may be ectopic or overexpression resulting from the transgene, and expression of NRX-1(γ) was notably higher than either endogenous or transgenic NRX-1(α) tagged alone. Broad endogenous expression of both isoforms of *nrx-1* precluded analysis of NRX-1(γ) specific expression in single neurons.

To further test whether the α isoform of *nrx-1* contributes to the behavioral response to food deprivation, we analyzed two *nrx-1* mutant alleles that specifically eliminate the α isoform (*nu485* and *gk*) (Fig. 1A). We found no difference in activity in α isoform-specific *nrx-1* mutants during the first hours of food deprivation compared to controls, but a significant reduction in activity during later hours (Fig. 2E, F, Suppl. Fig. 2C, D, Suppl. Fig. 3C). This result complements the behavioral phenotype observed when expressing NRX-1(α) in neurons, suggesting neuronal NRX-1(α) primarily mediates maintenance of increased activity off food. The α isoform-specific mutants have increased activity off food compared with selective pan-neuronal expression of NRX-1(γ)(Fig. 2C), so we asked if there was any role for NRX-1 in other tissues. We created a neuron-specific NRX-1(α) knockout by combining degradation of endogenous NRX-1 (all isoforms) in neurons (*rgef-1p::tir1*) with transgenic expression of NRX-1(γ) in neurons (*ric-19*p). This led to reduced activity off food only in the late hours, similar to the α isoform-specific *nrx-1* mutants (Fig. 2G), confirming that the γ isoform mediates initiation, likely functioning in neurons and other tissues (likely muscles). Therefore, both isoforms of *nrx-1* cooperate to initiate and maintain the behavioral response to food deprivation.

### The γ isoform of *nrx-1* contributes to octopamine signaling

The response to food deprivation is mediated in part by the monoamine neurotransmitter octopamine that is released from the RIC interneuron pair [41, 42]. To analyze the role of octopamine in our food deprivation assay, we tested mutants of the *tbh-1* gene which encodes the anabolic enzyme that creates octopamine. *tbh-1* mutants had significantly reduced responses to food deprivation compared to controls during only the first 2 h of food deprivation (Fig. 2H), suggesting that like NRX-1(γ), octopamine signaling contributes to the initial, but not maintained, responses to food deprivation. To test if *nrx-1* and *tbh-1* act in the same pathway, we generated *nrx-1(wy778);tbh-1* double mutants, which had significantly lower activity off food than controls for all 8 h (Fig. 2I). When compared to *nrx-1(wy778)* mutants, the double mutants had higher activity levels during the first 3 h of food deprivation, but no difference at later hours (Suppl. Fig. 1G, H). We also tested *nrx-1(gk);tbh-1* double mutants, which have reduced activity off food at both later hours of food deprivation like *nrx-1(gk)* single mutants, but also reduced activity during the first hours of food deprivation like *tbh-1* single mutants (Fig. 2J). These results further establish the link between octopamine signaling and initial response to food deprivation while highlighting a genetic interaction between octopamine and *nrx-1*, specifically a role for NRX-1(γ) in octopamine signaling.

**Fig. 3:**
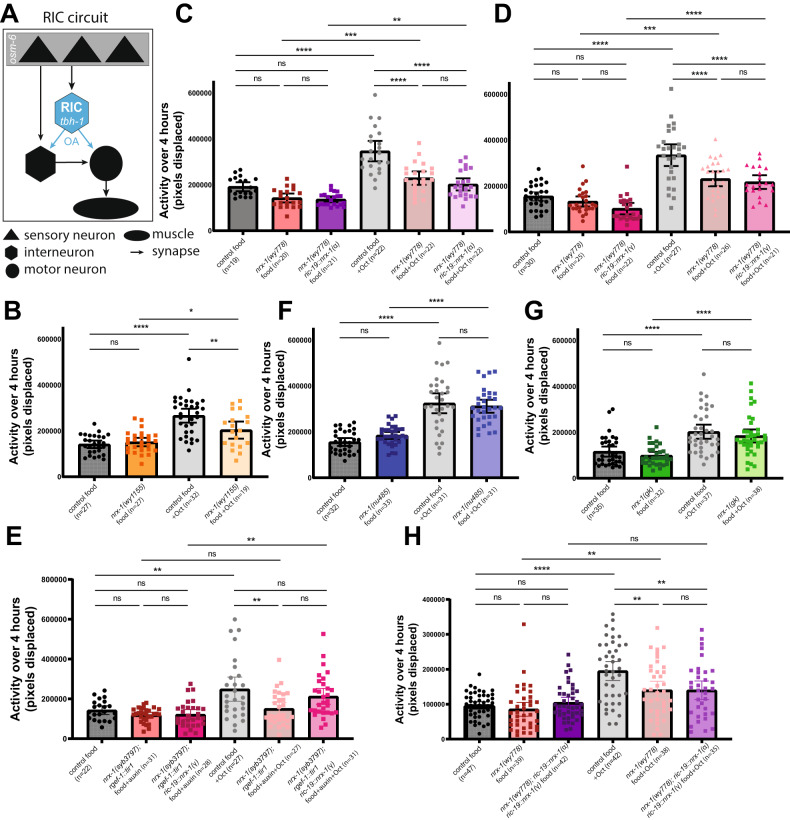
*nrx-1* is required for the response to exogenous octopamine. **A** Schematic of RIC octopaminergic circuit showing sensory neurons (triangles) projecting onto the RIC neurons (blue hexagon) and other interneurons (hexagons), which project onto motor neurons and muscles (circles/oval). Arrows indicate chemical synapses between neurons, and blue arrows represent potential octopamine signaling from RIC neurons. Average activity of day 1 adult control worms with food (black) or with food and 25 mM octopamine (gray) over 4 h compared with (**B**) *nrx-1(wy1155)* mutants (dark orange = food, light orange = food and 25 mM octopamine), (**C**) *nrx-1(wy778)* mutants (dark rose = food, light rose = food and 25 mM octopamine) and *nrx-1(wy778)* mutants expressing α isoform of NRX-1 in all neurons (*ric-19* promoter)(dark purple = food, light purple = food and 25 mM octopamine), (**D**) *nrx-1(wy778)* mutants (dark rose = food, light rose = food and 25 mM octopamine) and *nrx-1(wy778)* mutants expressing γ isoform of NRX-1 in all neurons (dark pink = food, light pink = food and 25 mM octopamine). (**E**) Average activity of day 1 adult worms with AID and sfGFP tagged NRX-1 expressing TIR1 in neurons without auxin (black with food, gray with food and 25 mM octopamine), with auxin (dark rose with food, light rose with food and 25 mM octopamine), and with auxin and expression of the γ isoform of NRX-1 in all neurons (*ric-19* promoter)(dark purple with food, light purple with food and 25 mM octopamine). **F** *nrx-1(nu485)* mutants (dark blue = food, light blue = food and 25 mM octopamine) (**G**) *nrx-1(gk)* mutants (dark green = food, light green = food and 25 mM octopamine). **H** Average activity of day 1 adult control worms with food (black) or with food and 25 mM octopamine (gray) over 4 h compared with *nrx-1(wy778)* mutants expressing α and γ isoforms of NRX-1 in all neurons (*ric-19* promoter)(dark purple = food, light purple = food and 25 mM octopamine). Individual points representing activity over 4 h are plotted and the bar and error bars indicate the mean and 95% CI. Results of the One-way ANOVA with Tukey HSD post hoc test: ns *p* > 0.05, *p < 0.05, **p < 0.01, ***p < 0.001, ****p < 0.0001.

We next analyzed the behavioral response of *nrx-1* mutants to exogenous octopamine. We found that exogenous octopamine significantly increased the activity of controls, even in the presence of food (Fig. 3A–H) [41]. The behavioral response to exogenous octopamine was significantly reduced in the *nrx-1* null alleles (*nrx-1(wy1155)* and *nrx-1(wy778))*(Fig. 3B–D) and following endogenous NRX-1 degradation in neurons (Fig. 3E), suggesting that neuronal NRX-1 is required for the response to octopamine. Interestingly, transgenic neuronal expression of NRX-1(α), NRX-1(γ), or both isoforms together did not restore the response to octopamine (Fig. 3C, D, H), indicating neuronal NRX-1 is not sufficient for responses to octopamine. α-isoform-specific *nrx-1* mutants (*nrx-1(nu486)* and *nrx-1(gk))* did respond to exogenous octopamine, confirming the specific requirement of NRX-1(γ) in octopamine signaling (Fig. 3F, G). Animals with neuron-specific NRX-1(α) knockout (degradation of NRX-1 in neurons with expression of NRX-1(γ) in neurons) partially responded to octopamine (Fig. 3E), further supporting a role for NRX-1(γ) in neurons and other cells. Degradation of NRX-1 in neurons did not impact activity on food (Fig. 3E).

### Expression of NRX-1(γ) in RIC neurons can restore the response to food deprivation but not octopamine

**Fig. 4:**
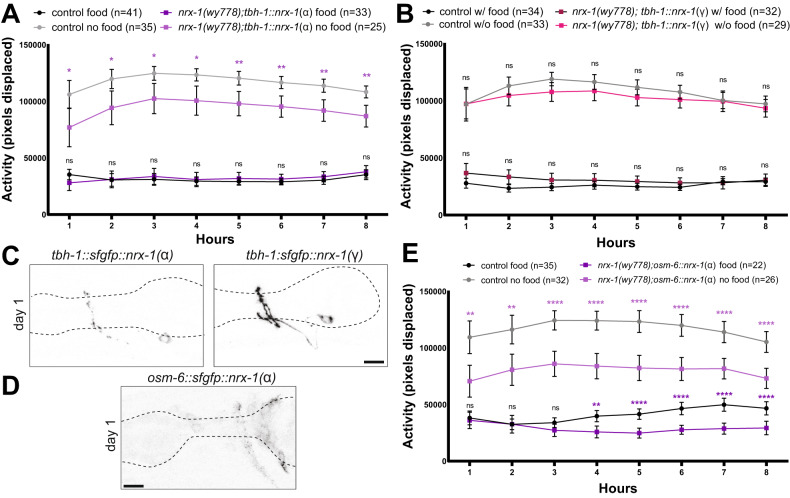
The γ isoform of NRX-1 in RIC neurons restores the behavioral response to food deprivation. Average activity of day 1 adult control worms in the presence (black) or absence of food (gray) compared to the average activity of (**A**) *nrx-1(wy778)* mutants expressing NRX-1(α) in the RIC neuron pair (*tbh-1* promoter)(dark purple with food, light purple without food) and (**B**) *nrx-1(wy778)* mutants expressing NRX-1(γ) in the RIC neuron pair (*tbh-1* promoter)(dark pink with food, light pink without food). Confocal micrographs of (**C**) sfGFP-tagged α (left) or γ (right) NRX-1 in the RIC neuron pair using the *tbh-1* promoter or (**D**) sfGFP-tagged NRX-1(α) in sensory neurons using the *osm-6* promoter in adult *nrx-1(wy778)* mutants (pseudo-colored black/white)(scale bar = 10 μm and dashed lines show outline of pharynx as a landmark). **E** Average activity of day 1 adult control worms in the presence (black) or absence of food (gray) compared to the average activity of *nrx-1(wy778)* mutant worms expressing the NRX-1(α) in sensory neurons (*osm-6* promoter)(dark purple with food, light purple without food). Means are plotted with 95% confidence intervals. Results of the Two-way ANOVA with Tukey HSD post hoc test: ns *p* > 0.05, *p < 0.05, **p < 0.01, ***p < 0.001, ****p < 0.0001.

The circuit controlling the behavioral response to food deprivation includes sensory neurons that project onto the octopaminergic RIC interneurons, which synapse onto other inter- and motor neurons to increase activity (Fig. 3A) [41, 53, 54]. We expressed α or γ NRX-1 tagged at the N-terminus with sfGFP in RIC neurons using the *tbh-1* promoter to test if either isoform in RIC is sufficient for either response to food deprivation [49, 55, 56]. *nrx-1(wy778)* mutants expressing NRX-1(α) in RIC had a reduced response to food deprivation for 8 h compared to controls (Fig. 4A, C, Suppl. Fig. 4A); however, *nrx-1(wy778)* mutants expressing NRX-1(γ) in RIC neurons had no differences in activity off food compared to controls (Fig. 4B, C, Suppl. Fig. 4B). This surprising behavioral rescue conflicts with the limited rescue observed with γ isoform in all neurons during food deprivation. Strong overexpression of NRX-1(γ) in RIC neurons (Fig. 4C) may be sufficient to alter the behavioral response through imbalance of *nrx-1* isoform expression across the circuit altering structure or function of synaptic connections. We previously found overexpression of NRX-1(γ) in a single neuron can induce gain of function phenotypes [37]. Expression of either isoform in RIC did not restore responsiveness to octopamine (Suppl. Fig. 4C, D), indicating the behavioral changes in responses to food deprivation resulting from the overexpression of NRX-1(γ) are not acting via octopamine response. Expression of NRX-1(α) in sensory neurons presynaptic to RIC (*osm-6*p)(Fig. 3A, Fig. 4D) had no effect off food and lowered activity on food compared to controls (Fig. 4E). These surprising results further suggest that imbalance of *nrx-1* isoform expression can result in behavioral changes distinct from loss of one or both isoforms more broadly and leave open where NRX-1(α) functions in the response to food deprivation.

### NRX-1(α) is required for RIC pre-synaptic and active zone morphology

**Fig. 5:**
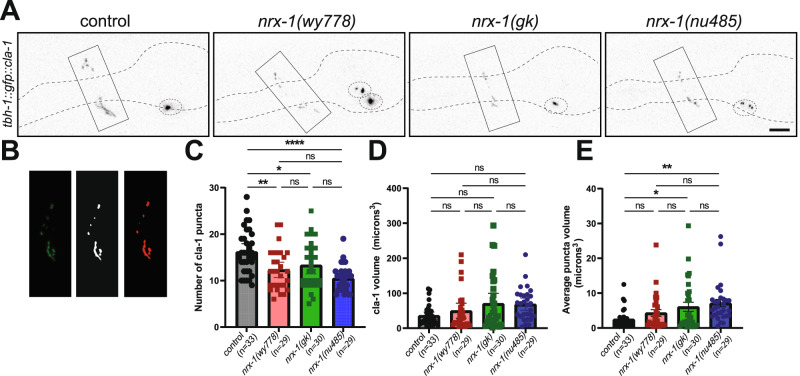
RIC neuron pre-synaptic puncta are reduced in α-specific *nrx-1* mutants. **A** Representative confocal micrographs of presynaptic puncta in RIC neurons visualized with *cla-1* tagged with gfp (*tbh-1* promoter) in adult control (left), *nrx-1(wy778)*(middle left), *nrx-1(gk)*(middle right), and *nrx-1(nu485)*(right) *C. elegans* (pseudo-colored black/white)(scale bar = 10 μm, dashed lines show outline of pharynx as a landmark, dashed ovals show RIC neuron soma, solid box outlines puncta in nerve ring). **B** Example image processing flow where the neurite of the RIC projecting into the nerve ring is cropped (left), the image thresholded in FIJI (middle), and then FIJI’s 3D object analyzer identifies individual objects (right). Comparison of (**C**) number of CLA-1 puncta (**D**) volume of CLA-1 puncta and (**E**) average volume of CLA-1 puncta. Black indicates control worms, red indicates *nrx-1(wy778)* mutants, green indicates *nrx-1(gk)* mutants, and blue indicated *nrx-1(nu485)* mutants. Means are plotted with 95% confidence intervals. Results of the One-Way ANOVA with Tukey HSD post hoc test: ns *p* > 0.05, *p < 0.05, **p < 0.01, ***p < 0.001, ****p < 0.0001.

We next asked whether NRX-1 has roles in pre-synaptic structure of the RIC octopamine neurons. We expressed a GFP-tagged CLA-1 [35, 36] using the *tbh-1* promoter to visualize presynaptic active zones of the RIC neurons (Fig. 5A, Suppl. Fig. 4E). We quantified RIC pre-synaptic morphology using 3D object analysis (Fig. 5A, B) and found that *nrx-1* mutants lacking both α and γ, or just α, *nrx-1* had reduced numbers of RIC pre-synaptic puncta relative to controls (Fig. 5C). There was no difference in total volume of puncta between controls and *nrx-1* mutants, but the α-isoform specific *nrx-1* mutants had increased average puncta volume (Fig. 5D, E). This demonstrates a specific role for NRX-1(α) in the presynaptic organization of the RIC neuron, which may impact release of octopamine, neuropeptides, or other neurotransmitters/signaling modalities from RIC. *nrx-1* has roles in pre-synaptic morphology and function, and this result supports the hypothesis that *nrx-1* function at synapses is isoform, neuron, and context dependent [35].

### Maintenance of the response to food deprivation by NRX-1(α) is dependent on *nlg-1*

**Fig. 6:**
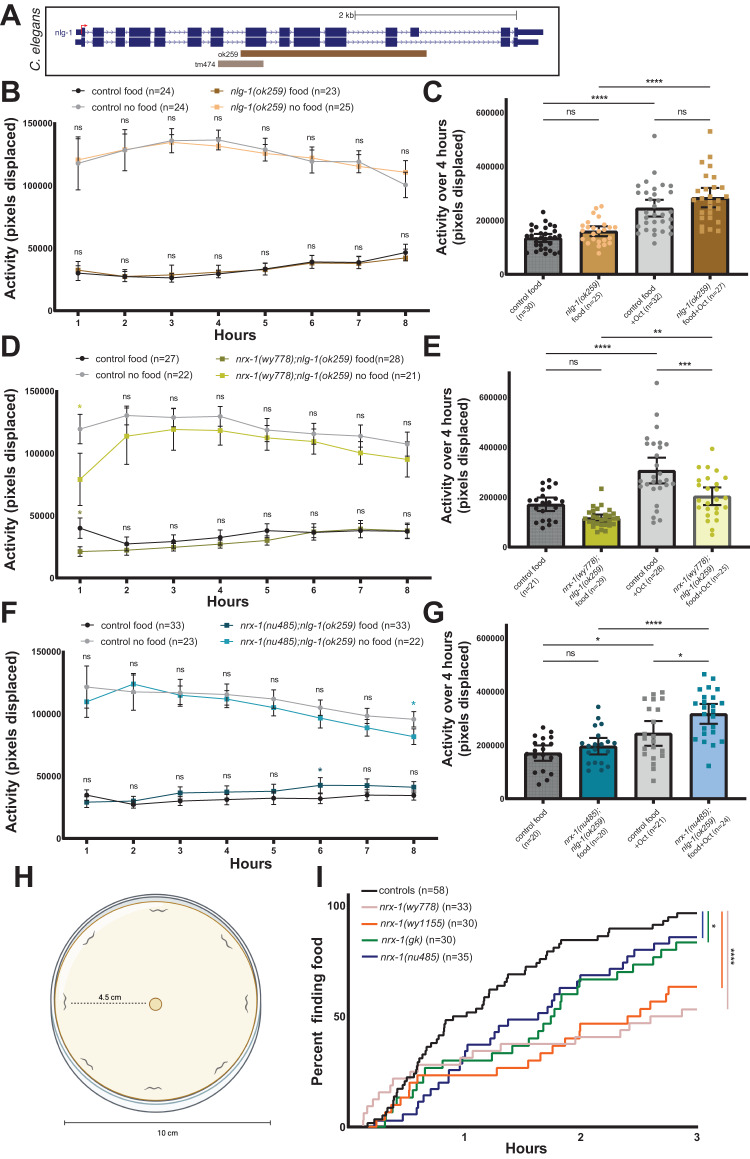
*nlg-1* and α isoform of *nrx-1* interact in maintenance of the behavioral response to food deprivation. **A** Schematic of *C. elegans nlg-1* genomic region and deletion alleles labeled. Average activity of day 1 adult control worms in the presence (black) or absence of food (gray) compared to the average activity of (**B**) *nlg-1(ok259)* mutants (dark brown with food, light brown without food), (**D**) *nrx-1(wy778); nlg-1(ok259)* double mutants (dark yellow with food, light yellow without food), (**F**) *nrx-1(nu485)*;*nlg-1(ok259)* double mutants (dark blue with food, light blue without food). Means are plotted with 95% confidence intervals. Results of the Two-way ANOVA with Tukey HSD post hoc test are written in the text. Average 4 h activity of day 1 adult control worms with food (black) or with food and 25 mM octopamine (gray) compared with (**C**) *nlg-1(ok259)* mutants (light brown with food, dark brown with food and 25 mM octopamine), (**E**) *nrx-1(wy778);nlg-1(ok259)* mutants (dark yellow with food, light yellow with food and 25 mM octopamine), (**G**) *nrx-1(nu485);nlg-1(ok259)* mutants (dark blue with food, light blue with food and 25 mM octopamine). Individual points representing activity over 4 h via the number of pixels displaced are plotted and the bar and error bars indicate the mean and 95% CI. Results of the One-way ANOVA with Tukey HSD post hoc test: ns *p* > 0.05, *p < 0.05, **p < 0.01, ***p < 0.001, ****p < 0.0001. **H** Schematic of the food finding assay in which 8 worms are equidistantly placed 4.5 cm away from a bacterial food source in the center of a 10 cm plate. **I** The percentage of worms of each genotype finding the food source over time. log-rank (Mantel-Cox) test with Bonferroni adjusted α level of 0.0125. ns *p* > 0.0125, *p < 0.0125, ****p < 0.0001.

We next analyzed whether NLG-1, the canonical trans-synaptic partner of NRX-1 [30], is involved in the response to food deprivation. Using two *nlg-1* deletion alleles (Fig. 6A), we found no consistent difference in activity in response to food deprivation, in the presence of food, or to exogenous octopamine (Fig. 6B, C, Suppl. Fig. 5A, B), suggesting *nlg-1* has no role in the behavioral responses to food deprivation or exogenous octopamine. To test the genetic interaction between *nrx-1* and *nlg-1*, we used double mutants lacking *nlg-1* and both isoforms of *nrx-1*, which had significant reductions in activity only during the first hour of food deprivation compared to controls (Fig. 6D), demonstrating that the failure of NRX-1(α)-specific mutants to maintain activity during food deprivation is dependent on NLG-1. We also found that the *nrx-1(wy778);nlg-1(ok259)* double mutants had significantly lower response to exogenous octopamine compared to controls (Fig. 6E), indicating that initiation by NRX-1(γ) is not dependent on NLG-1.

Because the *nrx-1(wy778);nlg-1(ok259)* double mutants had reduced initiation, which we attribute to NRX-1(γ), we tested *nlg-1* and α-isoform-specific *nrx-1* double mutants and found no difference in activity compared to controls with or without food (Fig. 6F) and an increase in activity in response to exogenous octopamine (Fig. 6G). This further supports distinct contributions for *nrx-1* isoforms in initiation and maintenance of the behavioral response to food deprivation, which may be in part due to differences in the ability of each isoform to interact with NLG-1, either through direct binding or through indirect interactions. The initiation and octopamine response are dependent on the γ isoform of *nrx-1*, independent of *nlg-1*, while maintenance is dependent on the α isoform of *nrx-1* and is conditional on *nlg-1* being present.

### *nrx-1* isoforms are required for *C. elegans* to find food

To determine the ethological relevance of our finding that *nrx-1* isoforms differentially impact activity levels upon food deprivation, we tested the ability of controls and mutants to find food (Fig. 6H). While nearly all controls and most α isoform-specific *nrx-1* mutants found the food target during the 3-h assay, only about half of *nrx-1* null mutants found the food in the same time (Fig. 6I, Suppl. Fig. 5C, D). When comparing the percent of animals finding food over time, we found a significant difference between both *nrx-1* null and α isoform-specific *nrx-1* mutants and controls (Fig. 6I). These results further demonstrate *nrx-1* isoform-specific alterations in the behavioral response to food deprivation and hint at the ethological relevance of our findings for foraging.

## Discussion

An organism’s foraging response is conserved and requires the coordination of numerous properties of the nervous system. Using the behavioral response to food deprivation in *C. elegans*, we report here novel cooperative usage of the α and γ isoforms of neurexin/*nrx-1* that regulates a sustained behavior over time. We find the γ isoform plays a role in initiation of foraging activity, in part via octopaminergic signaling, while the α isoform plays a role in maintaining foraging activity dependent on the presence of neuroligin/*nlg-1* (Suppl. Fig. 6). We also find the α and γ isoforms of *nrx-1* contribute to different structural and functional properties of the octopaminergic RIC neuron synapses, with the α isoform contributing to presynaptic active zone structure, and the γ isoform contributing to the downstream responsiveness to octopamine (Suppl. Fig. 6). Our results also suggest that the selective expression of *nrx-1* isoforms across neurons in a circuit is sufficient to modify activity and food-related behavior. The behavior of *C. elegans* lacking all neurexin isoforms was different than loss of a single isoform, and expression of single isoforms in all neurons was different than expression of single isoforms in subsets of neurons.

The role of each *nrx-1* isoform in coordinating the response to food deprivation begins to elucidate molecular mechanisms of how worms activate a response to food deprivation. The involvement of the NRX-1(γ) in initiation of the response involves octopamine, uncovering a novel temporal mechanism and increased complexity by which octopamine contributes to food responses [41, 57]. Worms lacking NRX-1(γ) were slow to initiate a response to food deprivation, strikingly similar to worms that lack octopamine signaling altogether (*tbh-1* mutants). Loss of *nrx-1* and *tbh-1* did not have a further reduction in activity off food compared to either alone, suggesting that *nrx-1* functions early through octopamine signaling. Worms with no *nrx-1* responded less to exogenous octopamine, while those lacking only NRX-1(α) responded normally to exogenous octopamine. This indicates that NRX-1(γ) may be involved in the correct wiring of the octopaminergic circuits controlling this behavior, facilitating release and/or receptivity of octopamine. Surprisingly, expression of NRX-1(γ) in neurons did not restore the response to octopamine, indicating it is required in other tissues that express it, such as muscles or glia. Expressing γ NRX-1 in neurons with endogenous *nrx-1* in other tissues intact partially restored the octopamine response, supporting a neuronal and non-neuronal role. Neuropeptide (*egl-21*) and glutamate signaling (*eat-4)* genes are both expressed in RIC [49], indicating at least two candidate signaling systems that could also contribute, although perhaps acting broadly throughout sensory neurons and the circuit, and not just in RIC. These results demonstrate a novel role for NRX-1(γ) in octopamine signaling, which is specific to initiation and not maintenance of the behavior, the exact mechanism of which needs to be further investigated.

The role of NRX-1(α) in maintenance of the response to food deprivation, meanwhile, is independent of octopamine response and dependent on *nlg-1*. Interactions between the canonical trans-synaptic binding partners *nrx-1* and *nlg-1* mediate several aspects of synaptic function and structure, often functioning synergistically and leading to similar morphological, neurophysiological, and even behavioral [30–32, 36, 38]. However, *nrx-1* and *nlg-1* have independent phenotypes in some contexts [34, 35, 58], or even opposing antagonistic phenotypes [37, 38]. Here, we find an epistatic relationship between *nrx-1* and *nlg-1* in the maintenance of the behavior where loss of *nlg-1* alone had no impact but was required in *nrx-1*(α) mutants for the altered maintenance mutant phenotype. This genetic interaction indicates that NRX-1(α) may be necessary when NLG-1 is present for the sustained response to food deprivation. We hypothesize that in the absence of NRX-1(α), NLG-1 may not cluster or localize correctly and lead to inappropriate interactions with other synaptic partners. While this may or may not represent a physical interaction between NRX-1(α) and NLG-1, prior studies have shown *nrx-1* and *madd-4/punctin* can redundantly recruit postsynaptic NLG-1 to cluster GABAergic receptors. Further, *nrx-1* appeared to help facilitate interactions between MADD-4 and NLG-1 [32]. Thus, an imbalance of *nrx-1* isoform expression could lead to altered NLG-1 clustering and/or lead to NLG-1 having gain of function interactions with other partners. This could lead to alterations in the neuronal circuity that would otherwise not happen in the absence of *nlg-1* and *nrx-1*, where redundant mechanisms could facilitate function of the circuit.

The coordination of structural and functional properties of synapses is crucial for behavior, and we find a nuanced relationship between structure and function of octopaminergic RIC synapses on the response to food deprivation. NRX-1(α), which has punctate expression in the axons of the nerve ring, has a role in organizing the development, maintenance, or structure of RIC neuron pre-synaptic active zones. This is similar to the subtle synaptic changes observed upon loss of *nrx-1* in other neuronal contexts in *C. elegans* [30, 34, 35], and adds further evidence that neurexins are important for pre-synaptic structure [1, 6]. While expression of NRX-1(α) in RIC may restore presynaptic structural phenotypes, we would correctly predict it would not restore octopamine responses, due to lack of NRX-1(γ) for the downstream response to octopamine. Our data also predicts that maintenance of the response requires non-octopamine signaling from RIC or other neurons, and that NRX-1(α) may be required in these other neurons. NRX-1(γ) does not have a major role in RIC presynaptic active zone structure, in contrast to its role in pre-synaptic active zones of motor neurons [35], which supports distinct context and neuron specific synaptic roles for *nrx-1* isoforms. We find a role for the γ isoform in synapse function (i.e. octopamine responsiveness), which could involve clustering of pre- or post-synaptic calcium channels and receptors, recruiting vesicles, or modifying signaling complexes [31, 32, 35]. The distinct roles of the isoforms in RIC synaptic structure and function, demonstrate that *nrx-1* isoforms play distinct and cooperative roles in the properties of synapses and circuits to generate behavior.

Perplexingly, expression of the α or γ isoforms in subsets of neurons, particularly the RIC interneurons, altered behavior in unexpected ways when compared to broader neuronal expression. The behavioral differences we observe may be due to imbalance of *nrx-1* isoforms leading to gain of function mechanisms and novel interactions to modify behavior. This is supported by the expression of the γ isoform in RIC fully rescuing the behavioral response to food deprivation but not rescuing the response to octopamine, suggesting that selective expression of NRX-1(γ) in RIC increases behavioral activity in the absence of food via a mechanism independent of octopamine that could include neuropeptide or glutamate signaling. This surprising result could be due to strong overexpression of NRX-1(γ), which may modify neuronal or synaptic properties during or after development or the structure of the circuit itself. We previously found overexpressing NRX-1(γ) in a single GABAergic neuron modified the neuron morphology and likely synaptic connections [37]. Our results, including unexpected behavioral phenotypes when differentially expressing *nrx-1* isoforms across the circuit, highlight the ability of *nrx-1* isoforms to modify behavior perhaps circumventing or uncovering roles for *nrx-1* in activating or inhibiting other neurons to modulate activity levels.

In this study, we identified mechanisms by which a conserved autism and schizophrenia relevant gene, *NRXN1*/*nrx-1*, influences behavior. We identified a novel role for the *NRXN1*/*nrx-1* γ isoform, which is uniquely to *NRXN1*. Furthermore, our work uncovers intricate cooperation between neurexin isoforms within a neuronal circuit, where isoform expression across a circuit modifies behavior. Much of the behavioral research on neurexins focuses on loss of single neurexin genes or isoforms [9, 17–19], and often attributes behavioral changes to single neurexin genes or isoforms. Interpretation of knockdown or expression of neurexin genes and isoforms should consider the role of remaining neurexin genes and isoforms, which may be acting individually or in combination with the neurexin gene or isoform manipulated. These results have implications for human *NRXN1* and behavior, where the most common variation observed is heterozygous deletion of α isoform-specific exons [8], and where neurexin isoform diversity is hugely increased compared to *C. elegans*. In addition, we uncover a novel genetic and isoform interaction in behavior with the conserved gene, *nlg-1/NLGN3*, which is also associated with autism [59]. Neurexins are incredibly complex genes in their isoform usage, redundancy, and context dependent functions (neuron, synapse, and circuit), which is further highlighted by our work. In a small nervous system with a singular neurexin gene, individual isoforms can have distinct and cooperative roles in behavior across neuronal circuits. Future studies will be needed to carefully define how specific neurexin isoforms contribute to previously described roles of neurexins and interactions at synapses, neuronal circuits, and behaviors.

### Supplementary information

Supplemental figure legends

Supplemental Figure 1

Supplemental Figure 2

Supplemental Figure 3

Supplemental Figure 4

Supplemental Figure 5

Supplemental Figure 6

Supplementary Table 1

Supplementary Table 2

Supplementary Table 3

## Data Availability

All data that supports the findings of this study are represented in the article, tables, and figures, with all WorMotel replicates and n listed in Supplemental Table 3. All data, plasmids, and strains are available from the corresponding author upon request.

## Materials availability

All data, plasmids, and strains are available from the corresponding author upon request.
