# Supplementary material for: Distinct neurexin isoforms cooperate to initiate and maintain foraging activity
Source: Transl Psychiatry. 2023 Nov 30;13:367. doi: 10.1038/s41398-023-02668-z (PMC10689797; doi:10.1038/s41398-023-02668-z)
Supplement: Supplementary file 1 — Supplemental figure legends [file 41398_2023_2668_MOESM1_ESM.docx]

**Supplementary Table 1. *C. elegans* strains by Figure**

**Supplementary Table 2. Plasmids and cloning information**

**Supplementary Table 3. Summary of replicates by figure and panel**

**Supplemental Figure 1. Normalized activity of *nrx-1* mutants and α NRX-1 in all neurons off food. A)** Image of a 48-well WorMotel taken on WormWatcher imaging platform with black and white inverted for visualization. WorMotel filled with agar and loaded with worms in which the top half of the wells contain food and the bottom half of the WorMotel lacks food. Representative zoom images of individual wells showing worms with and without food. **B)** Comparison of activity of controls, *nrx-1(wy778),* and *nrx-1(wy1155)* mutants normalized to experimental controls. Rose stars indicate a significant difference between *nrx-1(wy778)* and controls and orange stars indicate a significant difference between *nrx-1(wy1155)* and controls. **C)** Comparison of activity of N2 Bristol strain controls and worms expressing NRX-1 tagged with the AID and sfGFP on (black and dark purple respectively) and off (gray and light purple respectively) food. **D)** Comparison of activity between N2 Bristol strain controls off food when raised off (black) and on (gray) 4 mM auxin. **E)** Representative confocal micrographs of sfGFP and AID tagged NRX-1 with TIR1 expression in somatic cells (*etf-3::TIR1)* raised off (left) and on (right) 4mM auxin (pseudo-colored black/white). **F)** Comparison of activity of day 1 adult hermaphrodites with AID tagged NRX-1 expressing TIR1 in either all somatic cells (*eft-2::*TIR1) or all neurons (*rgef-1::TIR1)* raised on auxin normalized to experimental controls of the same genotype raised off auxin. Mean normalized activity is plotted with 95% confidence intervals. Results of the One-Way ANOVA with Tukey HSD or a Šidák post-hoc test: ns = p>0.05, * = p<0.05, ** = p<0.01, *** = p<0.001, **** = p<0.0001. **G)** Comparison of activity normalized to food deprived controls in controls (black), *nrx-1(wy778) (*rose), and *nrx-1(wy778);tbh-1* mutants (light blue). Light blue indicators next to control points indicate statistical differences between *nrx-1(wy778);tbh-1* mutants and controls and light blue indicators next to *nrx-1(wy778)* points indicate statistical differences between *nrx-1(wy778);tbh-1* mutants and *nrx-1(wy778)* mutants. **H)** Comparison of activity normalized to food deprived controls in controls (black), *nrx-1(wy778) (*rose), and *nrx-1(gk);tbh-1* mutants (light blue). Light blue indicators next to control points indicate statistical differences between *nrx-1(wy778);tbh-1* mutants and controls and light blue indicators next to *nrx-1(wy778)* points indicate statistical differences between *nrx-1(gk);tbh-1* mutants and *nrx-1(wy778)* mutants.

**Supplemental Figure 2. Distribution of activity values for *nrx-1* mutants.** Histograms showing the distribution of pixels displaced between consecutive images taken 10 seconds from each other on the food deprivation WorMotel set up for all four *nrx-1* alleles used in this study. **A)** *nrx-1(wy778)* in red, **B)** *nrx-1(wy1155)* in yellow, **C)** *nrx-1(nu485)* in blue, **D)** *nrx-1(gk)* in green. The distribution of the controls (N2 or OH15098) run in parallel with each *nrx-1* allele are in gray. Each graph represents (X) the distribution of worms with food for all hours, (x‘) worms without food for all hours of food deprivation, (x‘’) worms without food for the first hour of food deprivation, and (x‘’’) worms without food for the fifth hour of food deprivation.

**Supplemental Figure 3.** Comparison of activity normalized to food deprived controls in controls, *nrx-1(wy778)*, and **A)** *nrx-1(wy778)* mutants expressing the α isoform of *nrx-1* in all neurons (*ric-19* promoter) and **B)** *nrx-1(wy778)* mutants expressing the γ isoform of *nrx-1* in all neurons (*ric-19* promoter). Purple and pink indicators above the data indicate comparisons between controls and mutants expressing the α or γ isoform of *nrx-1* in all neurons. Purple and pink indicators below the data indicate comparisons between *nrx-1(wy778)* worms and *nrx-1(wy778)* mutants expressing the α or γ isoform of *nrx-1* in all neurons. **C)** Comparison of activity of controls, *nrx-1(nu485),* and *nrx-1(gk)* mutants normalized to experimental controls. Mean normalized activity is plotted with 95% confidence intervals. Results of the One-Way ANOVA with Tukey HSD post-hoc test: ns = p>0.05, * = p<0.05, ** = p<0.01, *** = p<0.001, **** = p<0.0001.

**Supplemental Figure 4: Normalized activity of *nrx-1(778)* mutants with RIC NRX-1 isoform expression off food.** Comparison of activity normalized to food deprived controls in controls, *nrx-1(wy778)* mutants, and **(A)** *nrx-1(wy778)* mutants expressing the α isoform of *nrx-1* in RIC neurons (*tbh-1* promoter) and **(B)** *nrx-1(wy778)* mutants expressing the γ isoform of *nrx-1* in RIC neurons (*tbh-1* promoter). Purple and pink indicators above the data indicate comparisons between controls and mutants expressing the α or γ isoform of *nrx-1* in RIC. Purple and pink indicators below the data indicate comparisons between *nrx-1(wy778)* worms and *nrx-1(wy778)* mutants expressing the α or γ isoform of *nrx-1* in RIC. Average activity of day 1 adult control worms with food (black) or with food and 25 mM octopamine (gray) over 4 hours compared with **(C)** *nrx-1(wy778)* mutants expressing α isoform of *nrx-1* in the RIC neuron pair (*tbh-1* promoter)(dark purple with food, light purple with food and octopamine) and **(D)** *nrx-1(wy778)* mutants expressing γ isoform of *nrx-1* in the RIC neuron pair (*tbh-1* promoter)(dark pink with food, light pink with food and octopamine). Results of the One-Way ANOVA with Tukey HSD post hoc test: ns = p>0.05, * = p<0.05, ** = p<0.01, *** = p<0.001, **** = p<0.0001. **E)** Representative confocal micrographs of CLA-1::GFP expressed in the RIC neurons in adult control (left), *nrx-1(wy778)* (middle), and *nrx-1(gk)* (right) C. elegans. RIC and RIM neuron morphology visualized with mCherry (*tdc-1* promoter) pseudo-colored red (top images) and RIC synaptic puncta visualized with *cla-1* tagged with gfp and pseudo-colored green (*tbh-1* promoter)(top and bottom)(scale bar = 10μm and dashed lines show outline of pharynx).

**Supplemental Figure 5. Activity of *nlg-1(tm474)* mutant allele and distribution of activity values for *nlg-1(ok259)*. A)** Average activity of day 1 adult control hermaphrodites in the presence (black) or absence of food (gray) compared with average activity of *nlg-1(tm474)* mutants (brown food, beige without food). Means are plotted with 95% confidence intervals. Results of the Two-Way ANOVA with Tukey HSD post hoc test: ns = p>0.05, * = p<0.05, ** = p<0.01, *** = p<0.001, **** = p<0.0001. **B)** Histograms showing the distribution of pixels displaced between consecutive images taken 10 seconds from each other on the food deprivation WorMotel set up for controls (gray) and *nlg-1(ok259*)(brown) on food and off food for all 8 hours. **C)** Latency of worms of each genotype to locate the food source. Worms not locating food for entire assay (3 hours) recorded as max time. Results of the One-Way ANOVA with Tukey HSD post hoc test: ns = p>0.05, * = p<0.05, ** = p<0.01, *** = p<0.001, **** = P<0.0001. **D)** The percentage of worms of each genotype that were able to successfully find the food in the good finding assay within 3 h. Pairwise Fisher’s Exact Test Bonferroni adjusted α level of 0.0125. ns *p* > 0.0125, **p* < 0.0125, *****p* < 0.0001.

**Supplemental Figure 6. Cooperation of multiple *nrx-1* isoforms in behavior**. In this study, we identified functional roles of *nrx-1* isoforms in the *C. elegans* response to food deprivation, which is a sustained increase in activity. The circuit controlling this response involves sensory neurons (green triangles) projecting onto interneurons (hexagons) including the pair of octopaminergic RIC interneurons (blue hexagons), which projects onto motor neurons (orange circles) and muscles (orange ovals). We find that the α, and largely uncharacterized γ, isoforms of *nrx-1* cooperate to initiate and maintain increased activity during food deprivation. The γ isoform is involved in the initiation response to food deprivation via octopamine signaling and is required in neurons (RIC) and in other tissues (such as muscles). The α isoform contributes to organization of presynaptic active zone structures involved in the release of octopamine and/or other neurotransmitters and neuropeptides from RIC, and other upstream neurons. The α isoform is involved in the maintained response to food deprivation that is conditional on whether the *nrx-1* canonical partner gene *nlg-1* is present. These results show a novel behavioral role of the γ isoform of *nrx-1* and show multiple isoforms of *nrx-1* act cooperatively to coordinate behavior.
