# Supplementary figures and images for: Distinct neurexin isoforms cooperate to initiate and maintain foraging activity
Source: Transl Psychiatry. 2023 Nov 30;13:367. doi: 10.1038/s41398-023-02668-z (PMC10689797; doi:10.1038/s41398-023-02668-z)

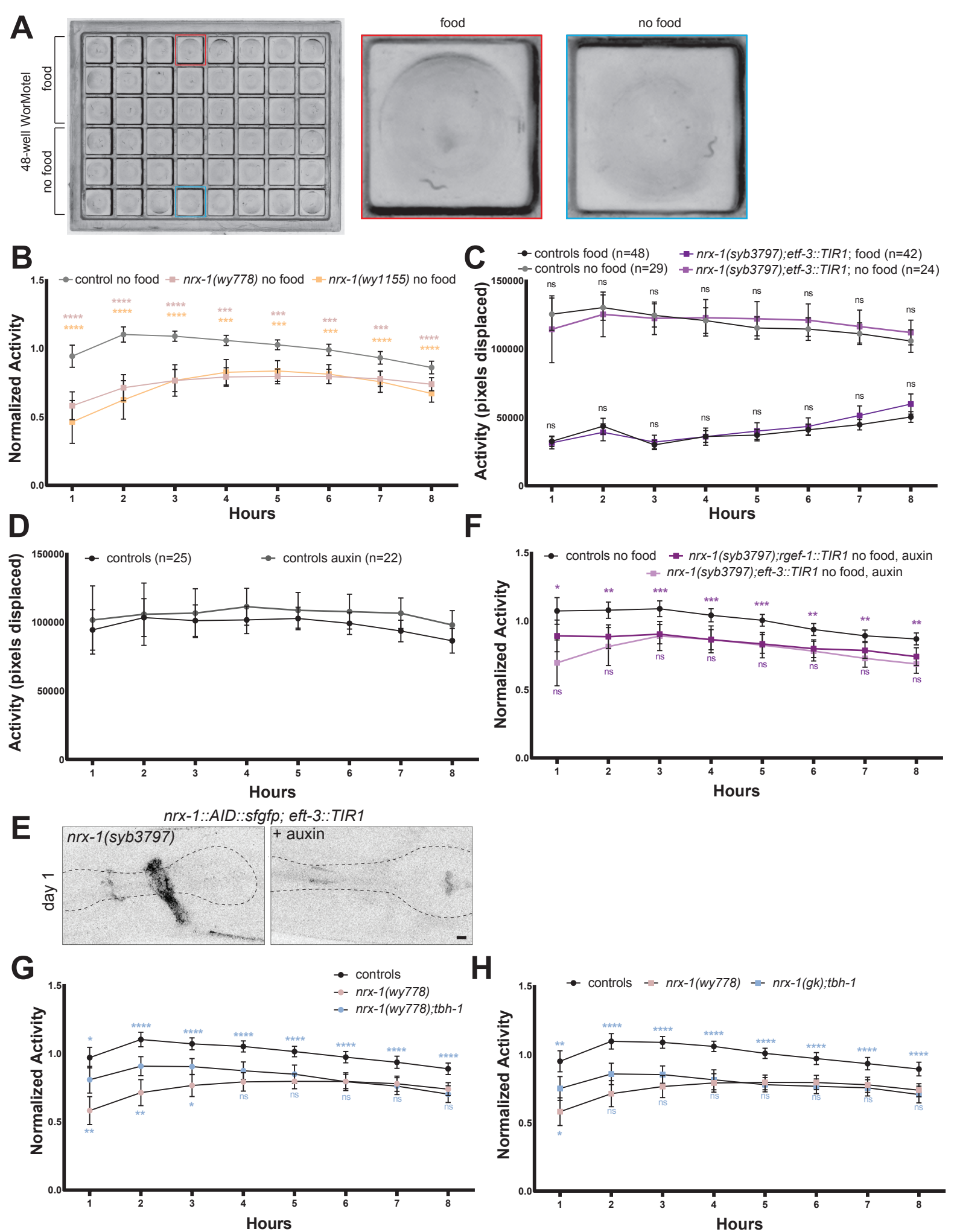

SUPPLEMENTAL FIGURE 1

Supplement: Supplementary file 2 — Supplemental Figure 1 [file 41398_2023_2668_MOESM2_ESM.pdf]

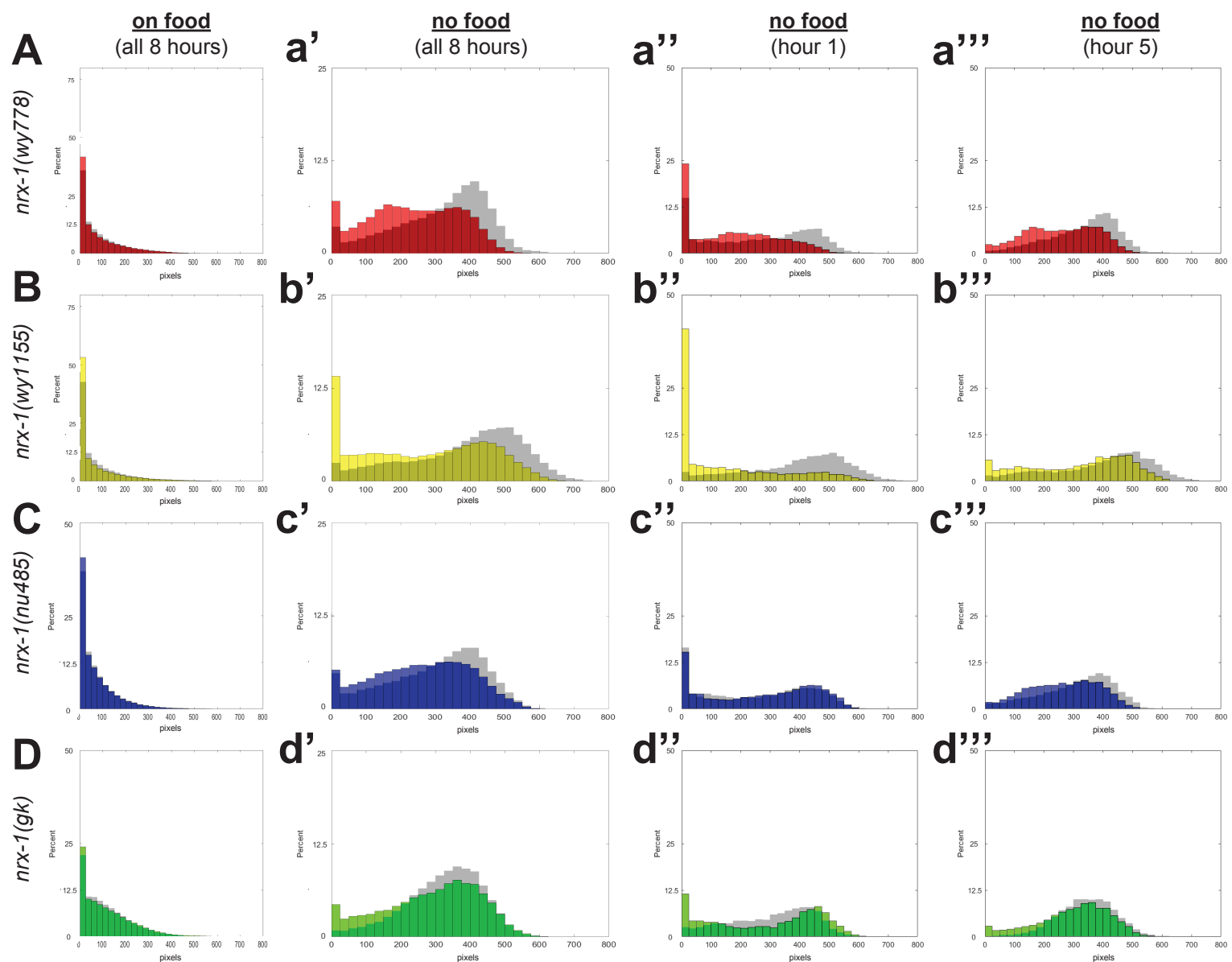

SUPPLEMENTAL FIGURE 2

Supplement: Supplementary file 3 — Supplemental Figure 2 [file 41398_2023_2668_MOESM3_ESM.pdf]

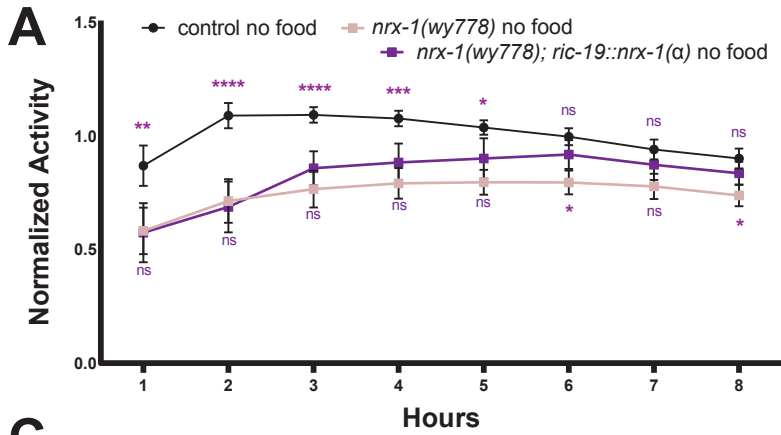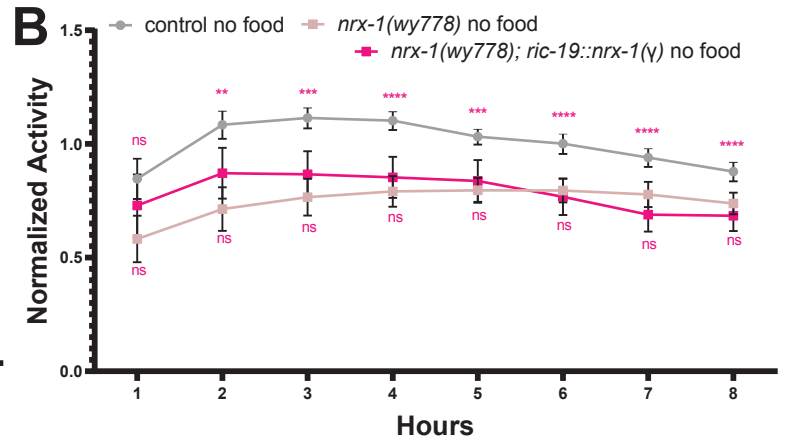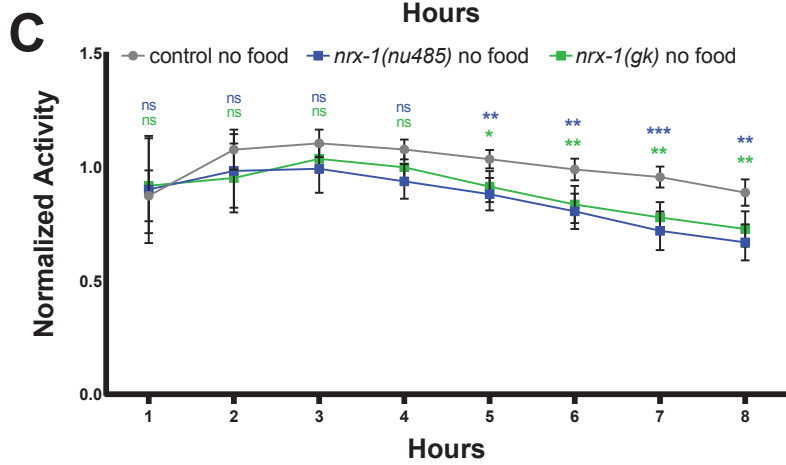

SUPPLEMENTAL FIGURE 3

Supplement: Supplementary file 4 — Supplemental Figure 3 [file 41398_2023_2668_MOESM4_ESM.pdf]

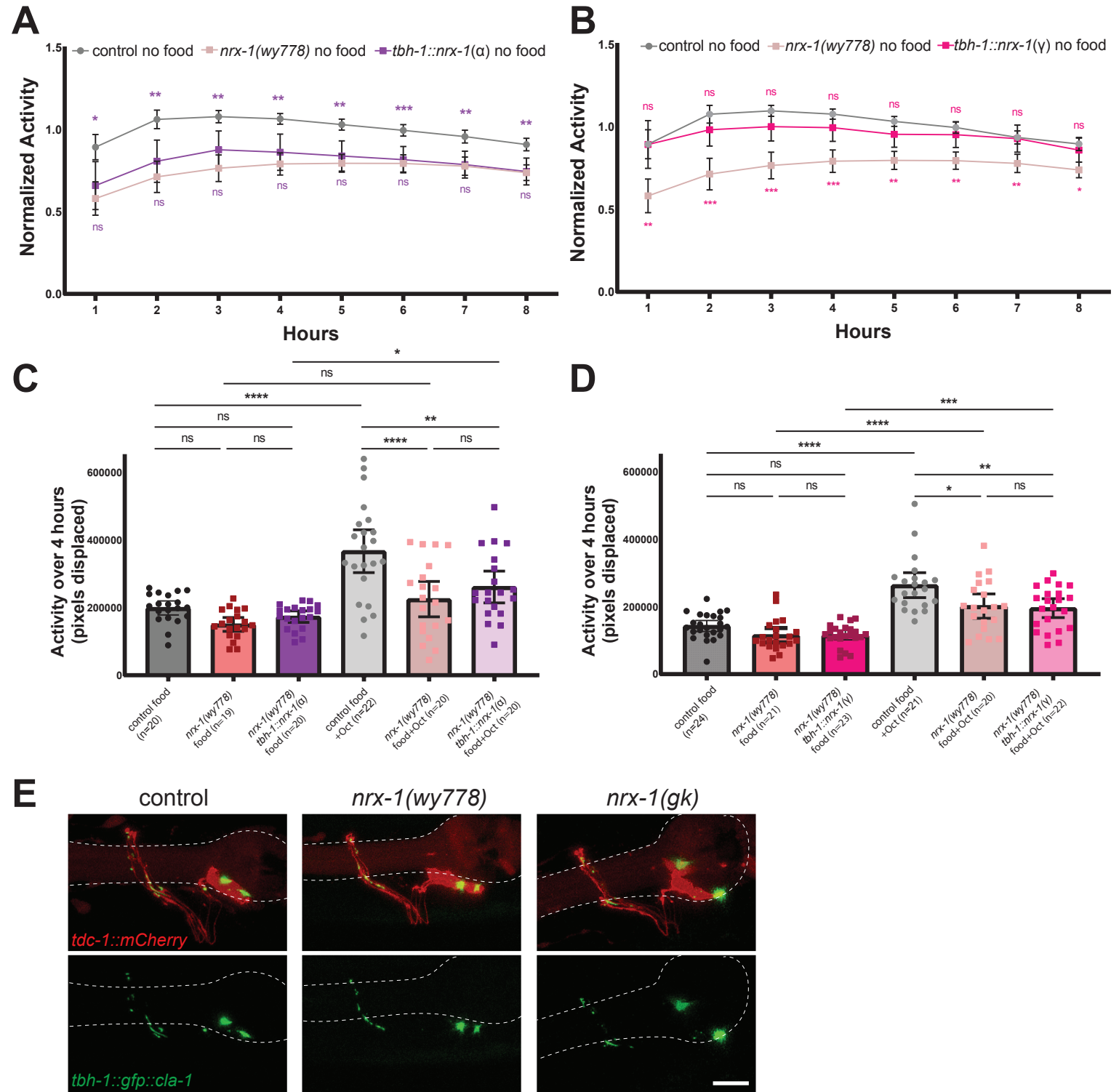

SUPPLEMENTAL FIGURE 4

Supplement: Supplementary file 5 — Supplemental Figure 4 [file 41398_2023_2668_MOESM5_ESM.pdf]

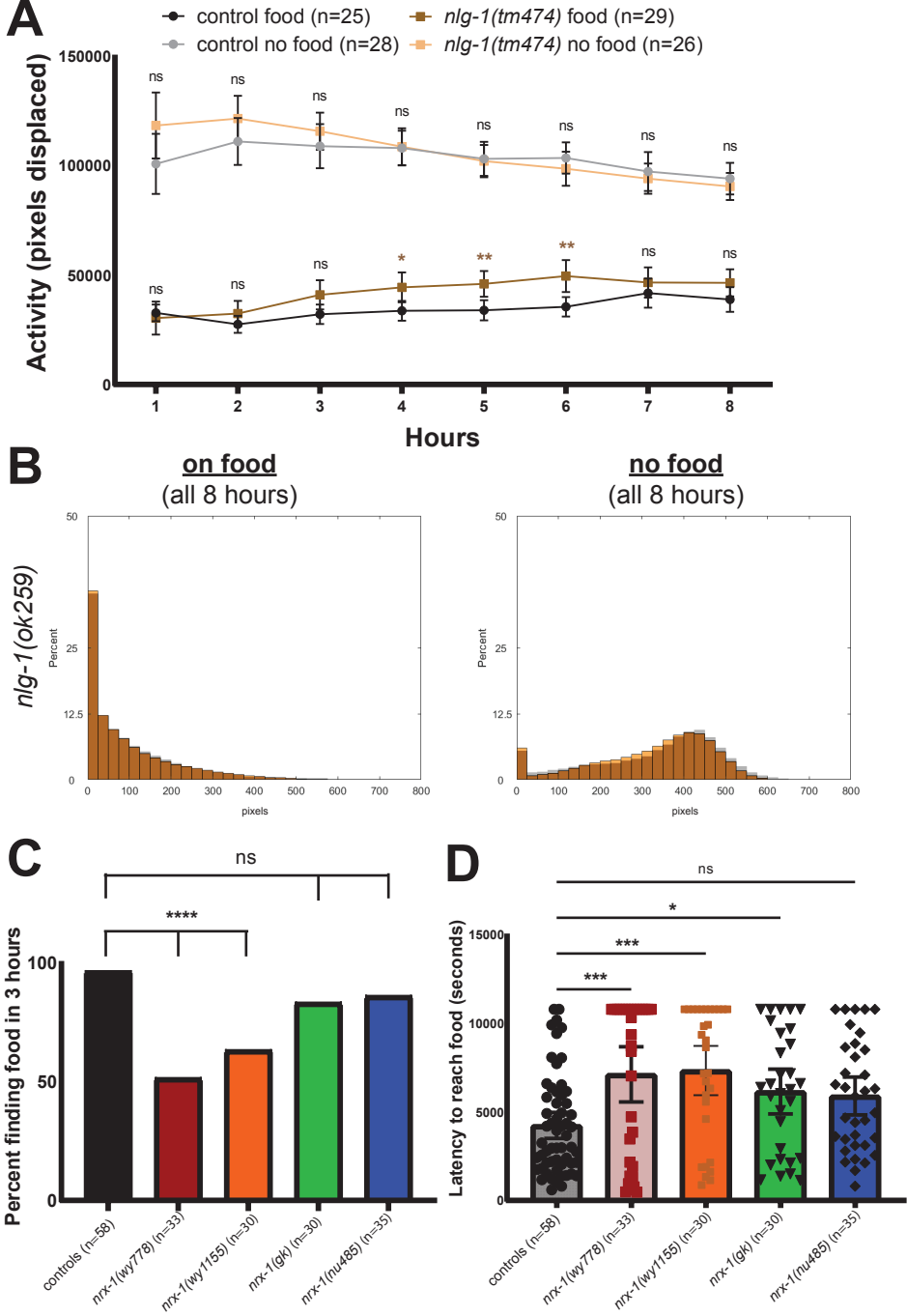

SUPPLEMENTAL FIGURE 5

Supplement: Supplementary file 6 — Supplemental Figure 5 [file 41398_2023_2668_MOESM6_ESM.pdf]

# Food Deprivation Response Behavior

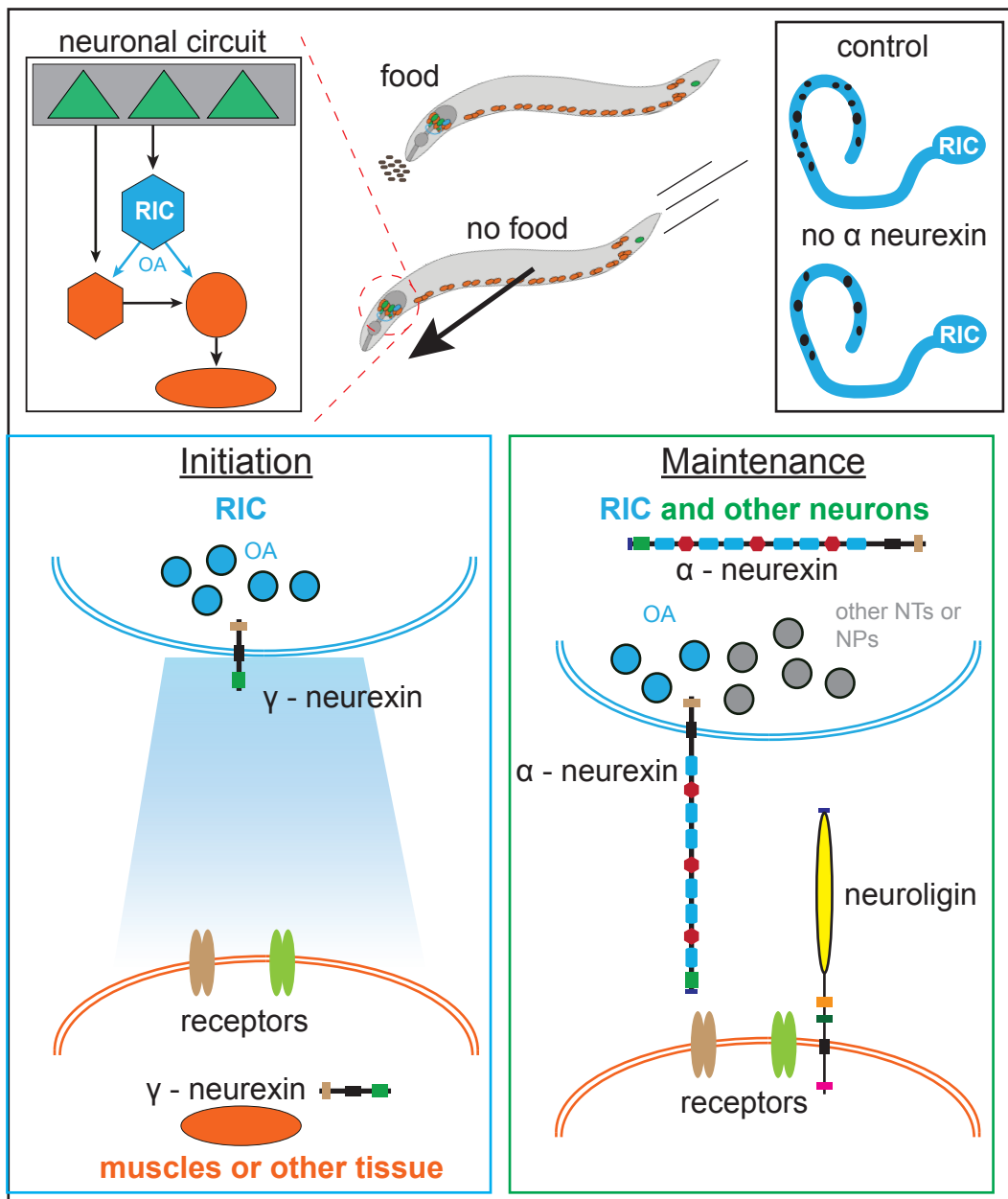

SUPPLEMENTAL FIGURE 6

Supplement: Supplementary file 7 — Supplemental Figure 6 [file 41398_2023_2668_MOESM7_ESM.pdf]
